# Are heart rate methods based on ergometer cycling and level treadmill walking interchangeable?

**DOI:** 10.1371/journal.pone.0237388

**Published:** 2020-08-06

**Authors:** Karin Olsson, Jane Salier Eriksson, Hans Rosdahl, Peter Schantz

**Affiliations:** Research Unit for Movement, Health and Environment, The Åstrand Laboratory and Laboratory of Applied Sport Science, The Swedish School of Sport and Health Sciences, GIH, Stockholm, Sweden; University of Calgary, CANADA

## Abstract

**Introduction:**

The heart rate (HR) method is a promising approach for evaluating oxygen uptake ($\dot{V}O_{2}$), energy demands and exercise intensities in different forms of physical activities. It would be valuable if the HR method, established on ergometer cycling, is interchangeable with other regular activities, such as level walking. This study therefore aimed to examine the interchangeability of the HR method when estimating $\dot{V}O_{2}$ for ergometer cycling and level treadmill walking in submaximal conditions.

**Methods:**

Two models of $\mathrm{HR}‐\dot{V}O_{2}$ regression equations for cycle ergometer exercise (CEE) and treadmill exercise (TE) were established with 34 active commuters. Model 1 consisted of three submaximal intensities of ergometer cycling or level walking, model 2 included also one additional workload of maximal ergometer cycling or running. The regression equations were used for estimating $\dot{V}O_{2}$ with seven individual HR values based on 25–85% of HR reserve (HRR). The $\dot{V}O_{2}$ estimations were compared between CEE and TE, within and between each model.

**Results:**

Only minor, and in most cases non-significant, average differences were observed when comparing the estimated $\dot{V}O_{2}$ levels between CEE and TE. Model 1 ranged from -0.4 to 4.8% (n.s.) between 25–85%HRR. In model 2, the differences between 25–65%HRR ranged from 1.3 to -2.7% (n.s.). At the two highest intensities, 75 and 85%HRR, $\dot{V}O_{2}$ was slightly lower (3.7%, 4.4%; P < 0.05), for CEE than TE. The inclusion of maximal exercise in the $\mathrm{HR}‐\dot{V}O_{2}$ relationships reduced the individual $\dot{V}O_{2}$ variations between the two exercise modalities.

**Conclusion:**

The HR methods, based on submaximal ergometer cycling and level walking, are interchangeable for estimating mean $\dot{V}O_{2}$ levels between 25–85% of HRR. Essentially, the same applies when adding maximal exercise in the $\mathrm{HR}‐\dot{V}O_{2}$ relationships. The inter-individual $\dot{V}O_{2}$ variation between ergometer cycling and treadmill exercise is reduced when using the HR method based on both submaximal and maximal workloads.

## Introduction

Acquiring knowledge of human oxygen uptake ($\dot{V}O_{2}$), energy demands and intensity levels in physical activities is valuable for health education and promotion as well as from research perspectives. This is since much of the understanding of exercise effects on weight control, morbidity, premature mortality, and physical performance is based on descriptions of intensity levels relative to the maximal oxygen uptake (e.g. [1, 2]), energy expenditure (e.g. [3, 4]) and MET hours (e.g. [5]). In these respects, the heart rate (HR) method can be a promising approach. It is based on the linear relationship between HR and $\dot{V}O_{2}$ during incremental loads of physical work under steady state conditions [6, 7]. Thanks to this linearity, $\dot{V}O_{2}$ can, in principle, be estimated by only measuring HR, and based on caloric coefficients [8, p. 104] the energy turnover can be calculated.

The first studies of the $\mathrm{HR}‐\dot{V}O_{2}$ relationship in humans were published in the early 1900s [9, 10]. Since then, several studies have used HR monitoring during various physical activities to understand energy requirements and exercise intensity in both humans [7, 11–14] and animals [15]. An accurate way of establishing $\mathrm{HR}‐\dot{V}O_{2}$ relationships is to create one for each individual, since there are large inter-individual variations, particularly due to sex, age, body weight and fitness level [16–19]. Today, these relationships are generally established in laboratories with portable HR monitors and stationary automated metabolic systems.

Even though the HR method has been used in several applied studies, a number of methodological issues connected to it appear not to have been sorted out completely, or only to a limited degree. These matters can be grouped into (1) reproducibility of $\mathrm{HR}‐\dot{V}O_{2}$ relationships and their outcomes when establishing the HR method under steady state conditions, (2) optimal ways of establishing $\mathrm{HR}‐\dot{V}O_{2}$ relationships under steady state conditions, (3) external validity of $\mathrm{HR}‐\dot{V}O_{2}$ relationships and the HR method established with one exercise form in relation to another, and (4) external validity of them in relation to both prolonged exercise at various constant exercise intensities as well as under non-steady state conditions.

It is only in recent times that fundamental methodological studies in terms of reproducibility have been carried out [20–22]. In general, these examinations have demonstrated good reproducibility at group level with minor relative differences between test and retest at an intensity range from resting to vigorous intensity levels. Several optimizing issues related to reproducibility and validity do however remain to be further explored. It is, for example, not known how many measuring points are needed, or how spread out they should be to enhance the reproducibility, and thereby the potential validity of the HR method.

As stated above, the interchangeability of the HR method between various forms of exercise to be investigated. So far, it is well known that dynamic exercise, with large muscle groups involved, has a different $\mathrm{HR}‐\dot{V}O_{2}$ relationship than static and dynamic exercise with smaller working muscle groups. For instance, $\dot{V}O_{2}$ has been found to be significantly lower during arm cycling compared to leg cycling as well as leg combined with arm cycling, when compared at equivalent HR values [23–25]. If the comparisons instead are limited to dynamic lower body work, through cycle ergometer and treadmill exercises, differences in the $\mathrm{HR}‐\dot{V}O_{2}$ relationships might not be obvious. When specifically comparing ergometer cycling with the simplest form of treadmill exercise, i.e. level walking, there is, to our knowledge, only one previous study. Berggren and Hohwü Christensen [26] observed similar $\mathrm{HR}‐\dot{V}O_{2}$ relationships for ergometer cycling compared to horizontal treadmill walking and running. The examination was performed on repeated occasions over a broad measurement range, containing a high number of submaximal steady state workloads for each of the three modalities. However, one considerable limitation was that only one subject, a well-trained man, was investigated.

The HR method can be applied principally in two different ways; one in which the oxygen uptake is measured, the other in which the oxygen uptake is estimated based on knowledge about the oxygen uptake at various workloads. For the latter purpose it is preferable to make use of a cycle ergometer. This is because oxygen consumption during steady state workloads of ergometer cycling is fairly constant between various individuals for both sexes [6] and independent of cycling experience [27]. Results reported by Åstrand [28] and Ryhming [29] demonstrated that two-thirds of healthy and trained individuals (50 men and 62 women) had $\dot{V}O_{2}$ levels within a range of ± 6% around mean levels at different workloads. Part of this variation can be explained by varying fitness levels, due to various relative positions of physiological thresholds [30–32], and varying muscle fibre type compositions [33]. However, a number of studies have shown that the oxygen cost at submaximal steady state work on cycle ergometers is also dependent on body weight [18, 34–39, p. 87]. Due to the global developments in body weight during the past three decades, this is an important factor to take into consideration when applying this form of exercise. If taking these determining factors into account, and considering that metabolic systems are expensive and technically complicated to use, especially in field exercise conditions [40, 41], it is advantageous that individual $\mathrm{HR}‐\dot{V}O_{2}$ relationships can be estimated with various cycle ergometer workloads. This would allow the HR method to be used in health education and promotion, when high measurement accuracy is not a decisive factor. It is also valuable from a research perspective in public health when large samples are to be investigated, which could be difficult to accomplish practically if metabolic measurement devices have to be used.

Considering the advantages in using the HR method, established through incremental constant workloads of ergometer cycling, it would be valuable if its $\mathrm{HR}‐\dot{V}O_{2}$ relationship and $\dot{V}O_{2}$ outcome when using the HR method mimic those of other common forms of dynamic exercise, such as walking. We have therefore investigated this issue further, with focus on the HR method’s interchangeability between submaximal ergometer cycling and level walking on a treadmill. At the same time, we have investigated if the HR method, based on ergometer cycling as well as treadmill exercise, can be methodologically optimized if endpoints of maximal exercise are added to the $\mathrm{HR}‐\dot{V}O_{2}$ relationships.

**Fig 1:**
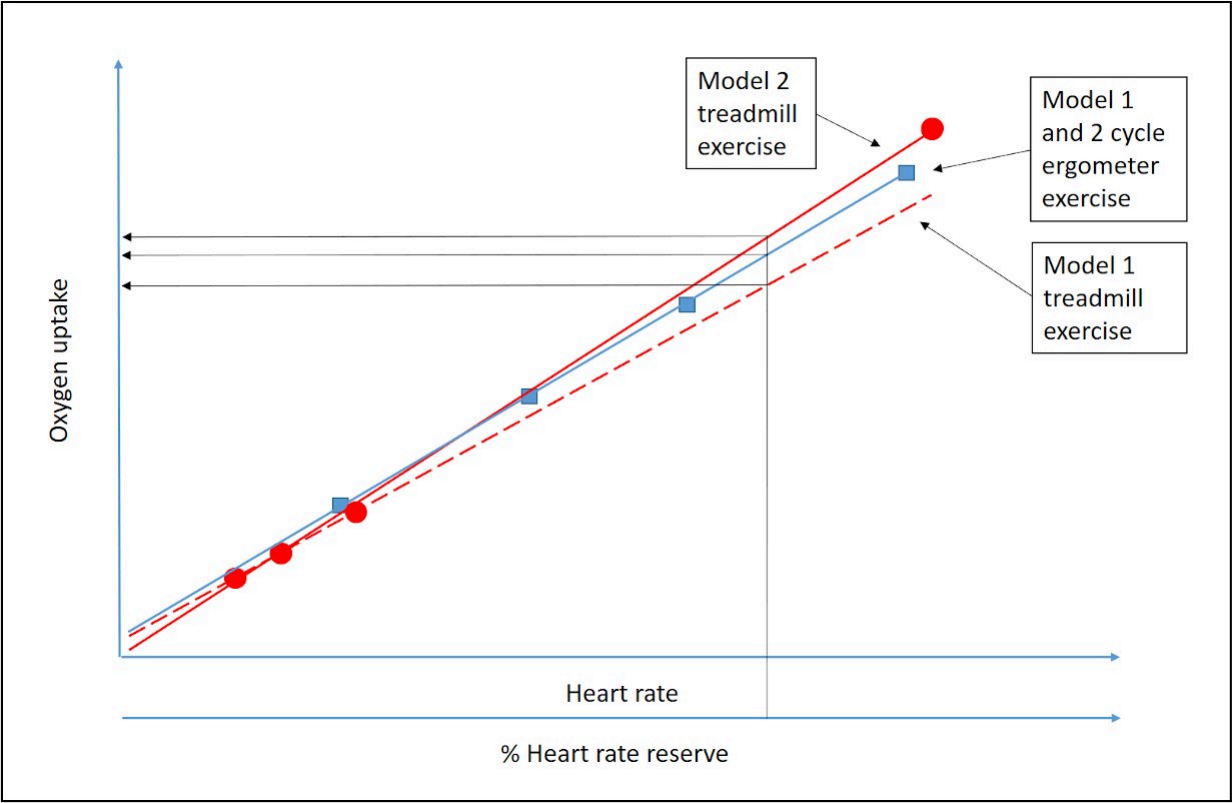
Principal scheme for the analytic models in the study. Two models are used for the HR method to estimate the oxygen uptake in cycle ergometer exercise versus treadmill exercise conditions, respectively. Model 1 is based on three submaximal workloads, whereas model 2 is based on the same three submaximal workloads as well as a workload corresponding to maximal exercise. The figure shows a conceivable example in which model 1 and model 2 in the treadmill exercise results in two different regression equations, whereas in the cycle ergometer exercise, model 1 and model 2 results in the same regression equation. The last step in the HR method is applied here in terms of oxygen uptake being estimated from various percentages of HR reserve and the different regression equations.

The aim of this study was therefore to examine the interchangeability of the HR method when estimating $\dot{V}O_{2}$ for ergometer cycling and level treadmill walking in submaximal conditions. In accordance with our previous reproducibility studies of the HR method [21, 22], two different models of $\mathrm{HR}‐\dot{V}O_{2}$ regression equations for cycle ergometer exercise (CEE) and treadmill exercise (TE), respectively, were constructed. Model 1 consisted of three submaximal steady state workloads of ergometer cycling or level walking, whereas model 2 also included one maximal workload of ergometer cycling or running. The $\mathrm{HR}‐\dot{V}O_{2}$ regression equations were applied to estimate oxygen uptake, using seven individually derived heart rate values based on % of heart rate reserve (%HRR). The outcomes from CEE and TE were then compared within and between each model (Fig 1).

## Methods

### Participants

Approval to conduct the study was obtained from the Ethics Committee North of the Karolinska Institute at the Karolinska Hospital (Dnr 03–637), Stockholm, Sweden.

#### Recruitment of participants

The recruitment of participants started by invitation through advertisements in two major morning newspapers in Stockholm. The inclusion criteria for participation were: being at least 20 years old, living in the County of Stockholm and cycling or walking the whole way, any distance, between home and place of work or study and actively commuting that way at least once a year. A questionnaire (The Physically Active Commuting in Greater Stockholm Questionnaire 1; PACS Q1, supporting information S1 Methods and S2 Methods) was sent to 2148 volunteers [42]. The recruitment of the participants in this particular study was based on those who had their age and commuting distance close to the median values of the overall project’s categories of female and male cyclists who only cycled to work (so-called single mode cyclists) and female and male pedestrians who only walked to work (so-called single mode pedestrians), respectively. They also rated their profession as very light or light physically. For details on the recruitment and categorization process as well as the questionnaire used, see Stigell and Schantz [42].

Information describing the physiological studies, tests and standardization procedures as well as a health declaration were sent to the selected volunteers. This information emphasized the right of the participants to cancel at any time during the study, without giving a reason. A signed informed consent of participation was returned. Based on the replies from the health declaration, individuals with high blood pressure or on medication that could affect the normal heart rate were excluded. The remaining were contacted until 10 individuals in each of the four categories (female and male single mode cyclists, and female and male single mode pedestrians) fulfilled the criteria. Due to the fact that there were no systematic differences between the cyclists and the pedestrians in this study's analyses, they were combined into one study group. Finally, with regard to some obstructive health conditions and technical problems with the equipment, there were 34 healthy and physically active commuters who had complete results; these are listed in Table 1.

**Table 1:** Descriptive characteristics of the participants (means ± standard deviations (SD)).

		Age	Height	Weight	BMI	HR_rest_
		(years)	(m)	(kg)	(kg·m^-2^)	(beats·min^-1^)
**Women**	**(n = 17)**	43 ± 4	1.70 ± 0.05	64 ± 9	22 ± 3	62.8 ± 7.9
**Men**	**(n = 17)**	45 ± 8	1.82 ± 0.06	82 ± 12	25 ± 3	66.7 ± 9.8
**All**	**(n = 34)**	44 ± 6	1.76 ± 0.08	73 ± 14	23 ± 3	64.7 ± 9.0

### Equipment

During all metabolic measurements, an automated stationary metabolic gas analysis system, Jaeger Oxycon Pro® (Carefusion GmbH, Hoechberg, Germany) was used in the mixing chamber mode. The software used was JLAB 4.53. The metabolic system was switched on 30 minutes before data collection and calibrated before and after each test using the automated procedures and according to the manufacturer’s recommendations. A high precision gas of 15.00% O_2_ and 6.00% CO_2_ (accuracy: O_2_ ± 0.04% and CO_2_ ± 0.1%, Air Liquid AB, Kungsängen, Sweden) was used for calibration. A face mask with a non-rebreathing air inlet valve (Combitox, Dräger Safety, Lübeck, Germany) was used, while a tube (inner diameter of 35 mm) was attached to the non-rebreathing valve to lead the exhaled air into the mixing chamber. The measured metabolic variables, including HR, were saved in average values of 15 seconds. Using the mixing chamber mode, Oxycon Pro has been found to be reliable (coefficient of variation for $\dot{V}O_{2}$: 1.2% between 0.5–6.0 L·min^-1^) [43] and valid against the golden standard method [41, 43]. The HR measurements were performed using Polar Electro S610i Heart Rate Monitor and the associated Polar Wearlink 31 transmitter (Polar Electro Oy, Kempele, Finland).

The cycle ergometer exercise was performed on a manually braked Monark pendulum ergometer cycle 828E (Monark Exercise AB, Vansbro, Sweden). Immediately before each test, the scale was zeroed while the subjects sat on the saddle with their feet resting on the surface between the pedals. A digital metronome (DM70 Seiko S-Yard Co. Ltd, Tokyo, Japan) was used to keep correct cycling cadence. At regular intervals of one minute, the work rate was controlled by checking the cadence and the braking force as indicated on the pendulum scale. The treadmill exercise was conducted on RL2500E treadmill ergometer (Rodby Innovation AB, Vänge, Hagby, Sweden). Its accuracy for velocity and inclination were: ± 1.8% and 0.2˚, respectively.

### Measurements

#### Study design and standardization

The subjects visited the laboratory on three different occasions. All occasions followed the same procedure, including measurements of HR at rest, simultaneous measurements of HR and $\dot{V}O_{2}$ at submaximal and maximal level, with the only difference between the occasions being the type of physical work; twice with cycle ergometer exercise (CEE) and once with treadmill exercise (TE). The first occasion always consisted of CEE for all subjects. For most of the subjects, CEE was also performed at session two and followed by TE at session three. The purpose of conducting repeated sessions with CEE was to familiarize the subjects with the experimental process during the first session (familiarization session). The number of days between the three occasions was 8 ± 9 days (means ± SD). Two educated investigators carried out the experimental tests, all participants had the same investigator for each occasion.

Prior to each test, the participants were instructed to follow some standardization guidelines: 1) not to take part in any vigorous exercise for 24 hours before the tests, 2) not to cycle to the laboratory, 3) to refrain from eating, drinking, smoking and taking snuff for at least one hour before arrival at the laboratory, 4) not to eat a large meal at least three hours before the tests, 5) to avoid stress and 6) to cancel the test if they had fever, an infection or a cold.

On arrival at the laboratory, measurements of body weight and height (only in the familiarization session) were conducted. Thereafter, a resting HR measurement was performed in supine position on a treatment table during ten minutes, and values from the last five minutes were used. Anthropometric and resting HR data were from the familiarization session, while HR and $\dot{V}O_{2}$ data during exercise were from sessions 2 and 3.

#### Cycle ergometer exercise

The submaximal ergometer cycling consisted of three different workloads: 50, 100 and 150 watt (W) for the female and 100, 150 and 200 W for the male participants. At each workload, the participants cycled until steady state occurred (approximately six minutes), after which the resistance was increased. The third workload was increased to only 125 W or 175 W for women and men respectively, if after the second workload, the subjects' HR was higher than 150 beats·min^-1^ and their rated perceived exertion (RPE), according to the Borg scale, exceeded 15 for both legs and breathing [44]. A cadence of 50 revolutions per minute (rpm) was used since it was when using this cadence that oxygen consumption has been found fairly constant between individuals [6, 28, p. 19, 29].

Between the second and third workload, the participants continued cycling for one minute at a self-selected low cadence with a resistance of 5 Newton (N). The subjects were then instructed to resume the cadence of 50 rpm while the investigator gradually increased the resistance until, after one minute, the third work rate was reached (resistance was increased to 50 W during the first 15 seconds, to 100 W the second 15 seconds and successively to the third work rate during the last 30 seconds). After the submaximal workloads, and before the maximal test, the participants continued cycling for two minutes at a self-chosen low cadence and a resistance of 5 N.

The maximal test was performed with a cycle cadence of 80 rpm. During the first three minutes, the work rates were set to 60, 100 and 120 or 140 W for one minute each. The latter alternatives depended on which third workload the subjects had achieved during the submaximal phase; 120 W was chosen if the third work rate had been 125 W or 175 W for women and men, respectively, and 140 W was chosen if it had been 150 W or 200 W for women and men, respectively. After these first three minutes, the resistance was increased by 20 W every continued minute until voluntary exhaustion occurred. The increments of work rate were chosen in accordance with our previous evaluation [21].

#### Treadmill exercise

The submaximal treadmill exercise consisted of three workloads of level (0.0˚) walking. Velocities were set to: 4.0, 4.9 and 5.9 km·h^-1^. The participants continued walking at each workload until steady state was attained (normally five minutes). Immediately after completing the third walking load, the speed was increased to a comfortable level for running, after which the maximal phase was started.

The maximal running test was performed through constant speed (9.0 ± 0.7 km·h^-1^) and successive increments of inclination. During the first minute of the test, the inclination was set to 0.0˚, after one minute it was increased to 1.0˚ and then by 0.5˚ every continued minute until voluntary exhaustion occurred and the test was terminated.

For both exercise modalities, rating of RPE for legs and breathing, respectively, was noted at the end of each submaximal workload as well as directly after completion of the maximal tests. In order to ensure that the cycling and running maximal tests achieved their purposes, at least two of the following three criteria were met by each participant: 1) a plateau in $\dot{V}O_{2}$ despite increasing exercise intensity (defined as a $\dot{V}O_{2}$ increment of < 150 ml), 2) a respiratory exchange ratio of ≥ 1.10 and 3) a RPE rating of ≥ 17 [45–47].

### Data processing and statistical analyses

For determination of HR at rest, values are based on each single time period between the heart rate beats, the average of the last five minutes was transformed to beats per minute. For all forms of submaximal exercise, paired HR and $\dot{V}O_{2}$ values during the last, of the two consecutive minutes, at steady state have been used as averages for each workload. Since HR has been found to be more variable than $\dot{V}O_{2}$ at lower intensity levels [48], steady state was defined when HR values were within 2–3 beats·min^-1^. As recommended by Howley, Bassett [46], maximal values were calculated by averaging the minute with highest continuous paired values of HR and $\dot{V}O_{2}$. For calculation of a certain percentage level of heart rate reserve (%HRR), both the resting and maximal heart rate values were used in the equation [49]. To exemplify, the HR value of interest was subtracted with HR_rest_ and then divided by HR_max_ minus HR_rest_. When the submaximal workloads for cycling are expressed in relative levels of HR_max_, HRR and $\dot{V}O_{2\max}$, these levels have been based on the values from the maximal cycling test. Conversely, the maximal values from the treadmill test have been used to describe the percentage levels for treadmill walking.

The $\mathrm{HR}‐\dot{V}O_{2}$ relationships, based on the individual paired values of HR and $\dot{V}O_{2}$ from the three submaximal workloads (model 1) plus one maximal workload (model 2), were described by linear regression analyses for the two forms of exercise within each model. The individual regression equations were then used by the HR method for estimating $\dot{V}O_{2}$. Individual HR values, based on % of HRR were created according to the American College of Sports Medicine’s classification of exercise intensity [50]. The intensity levels: 25 and 35%HRR, (very light to light exercise intensity), 45 and 55%HRR (moderate exercise intensity) and 65, 75 and 85%HRR (vigorous exercise intensity) were selected to cover a broad range of commonly used HR values during submaximal physical activities. In the calculations of individual HR values, HRR was always based on the maximal values from the running test. The individually derived HR values were then used in each individual regression equation for estimating $\dot{V}O_{2}$ at the seven intensity levels. Confidence intervals (CI) of 95% were calculated for the absolute values of the regression coefficients (y-intercept, slope and r-coefficient) and the $\dot{V}O_{2}$ estimations, as well as the differences between the modalities. Analyses of both the absolute and relative exercise mode differences were performed with Student´s one-sample t-test and Wilcoxon´s one-sample signed rank test at the group level for all participants (n = 34), since no systematic gender variations were observed.

The estimated mean $\dot{V}O_{2}$ levels for CEE and TE, respectively, based on all individual values between 25–85% of HRR, have been illustrated through linear line graphs with 95% CI for both models. Linear regression equations, including 95% CI, were created to exemplify the different $\mathrm{HR}‐\dot{V}O_{2}$ relationships. All individual estimated $\dot{V}O_{2}$ values were also graphically pairwise compared between the exercise modalities and models in scatter plots and regressions equations, including 95% CI. The linear line graphs and the scatter plots were created using Graph-Pad Prism® 8 software package (Graph-Pad Software Inc., San Diego, CA, USA). The other statistical analyses were performed using the Statistical Package for the Social Sciences (IBM SPSS Statistics, 25 and 26, Chicago, IL, USA). Values are presented as means ± SD, unless otherwise stated. An alpha level of P < 0.05 has been used in all analyses.

## Results

### Descriptive data on cycle ergometer and treadmill exercises

Descriptive data from the cycle ergometer exercise (CEE) and the treadmill exercise (TE) are presented for the female and male participants separately, and for all participants together in Tables 2 and 3. Mean values of $\dot{V}O_{2}$ and HR are expressed in both absolute and relative numbers for all workloads (three submaximal and one maximal) within each form of exercise. The submaximal HR levels for all participants, ranged from 103.3 ± 12.7 to 148.7 ± 11.9 beats·min^-1^ during CEE (Table 2) and from 89.1 ± 13.2 to 104.9 ± 12.7 beats·min^-1^ during TE (Table 3). The corresponding maximal HR values were 178.9 ± 9.2 and 181.8 ± 8.3 beats·min^-1^, respectively. For the $\dot{V}O_{2}$ and RPE levels of each workload and exercise modality, see Tables 2 and 3.

**Table 2:** $\dot{V}O_{2}$, HR, $\dot{V}O_{2}/\mathrm{HR}$ and RPE during submaximal and maximal cycle ergometer exercise (means ± SD).

	Workload	$\dot{V}O_{2}$			HR			$\dot{V}O_{2}/\mathrm{HR}$	RPE	
	W	L·min^-1^	mL·min^-1^·kg^-1^	$\%\dot{V}O_{2}{}_{\max}$	beats·min^-1^	%HR_max_	%HRR	L·min^-1^/ beats·min^-1^	legs	breathing
**Women**	**50**	0.83 ± 0.09	13.2 ± 1.6	34.2 ± 4.9	98.2 ± 8.2	54.9 ± 3.7	30.2 ± 5.3	0.0085 ± 0.0010	8.8 ± 1.4	8.8 ± 1.2
**(n = 17)**	**100**	1.40 ± 0.11	22.3 ± 2.8	57.7 ± 7.3	126.9 ± 9.5	70.9 ± 4.6	54.9 ± 7.7	0.0111 ± 0.0010	12.2 ± 1.8	12.1 ± 1.8
	**137 ± 13**	1.88 ± 0.19	29.7 ± 3.3	76.9 ± 7.4	151.1 ± 7.7	84.5 ± 3.8	75.9 ± 6.2	0.0125 ± 0.0014	15.3 ± 1.0	15.1 ± 1.3
	**Max**	2.46 ± 0.28	38.9 ± 4.5	100.0 ± 0.0	179.0 ± 8.0	100.0 ± 0.0	100.0 ± 0.0	0.0137 ± 0.0013	18.6 ± 1.2	18.7 ± 1.1
**Men**	**100**	1.43 ± 0.17	17.7 ± 2.7	43.5 ± 8.8	108.4 ± 14.6	60.5 ± 6.1	37.1 ± 8.4	0.0134 ± 0.0022	10.9 ± 1.8	10.5 ± 1.8
**(n = 17)**	**146 ± 11**	2.02 ± 0.24	24.9 ± 3.7	61.0 ± 10.0	129.1 ± 15.8	72.1 ± 6.8	55.7 ± 9.8	0.0159 ± 0.0026	13.5 ± 1.7	12.9 ± 2.2
	**179 ± 20**	2.46 ± 0.28	30.4 ± 4.7	74.0 ± 9.7	146.2 ± 14.9	81.8 ± 6.1	71.1 ± 8.9	0.0170 ± 0.0027	15.5 ± 1.5	14.4 ± 2.2
	**Max**	3.38 ± 0.55	41.4 ± 6.3	100.0 ± 0.0	178.7 ± 10.6	100.0 ± 0.0	100.0 ± 0.0	0.0190 ± 0.0033	18.3 ± 1.0	17.9 ± 1.9
**All**	**75 ± 25**	1.13 ± 0.33	15.4 ± 3.2	38.9 ± 8.5	103.3 ± 12.7	57.7 ± 5.7	33.7 ± 7.7	0.0110 ± 0.0030	9.9 ± 1.9	9.7 ± 1.8
**(n = 34)**	**123 ± 25**	1.71 ± 0.36	23.6 ± 3.5	59.4 ± 8.8	128.0 ± 12.9	71.5 ± 5.8	55.3 ± 8.7	0.0135 ± 0.0031	12.9 ± 1.9	12.5 ± 2.0
	**158 ± 27**	2.17 ± 0.38	30.1 ± 4.0	75.5 ± 8.6	148.7 ± 11.9	83.1 ± 5.2	73.5 ± 7.9	0.0148 ± 0.0032	15.4 ± 1.3	14.7 ± 1.8
	**Max**	2.92 ± 0.64	40.2 ± 5.5	100.0 ± 0.0	178.9 ± 9.2	100.0 ± 0.0	100.0 ± 0.0	0.0163 ± 0.0037	18.4 ± 1.1	18.3 ± 1.6

**Table 3:** $\dot{V}O_{2}$, HR, $\dot{V}O_{2}/\mathrm{HR}$ and RPE during submaximal (walking) and maximal (running) treadmill exercise (means ± SD).

	Workload	$\dot{V}O_{2}$			HR			$\dot{V}O_{2}/\mathrm{HR}$	RPE	
	km·h^-1^	L·min^-1^	mL·min^-1^·kg^-1^	$\%\dot{V}O_{2\max}$	beats·min^-1^	%HR_max_	%HRR	L·min^-1^/ beats·min^-1^	legs	breathing
**Women**	**4.0**	0.64 ± 0.09	10.0 ± 1.1	24.1 ± 3.3	87.8 ± 11.7	48.1 ± 6.5	21.0 ± 6.3	0.0073 ± 0.0009	7.5 ± 1.2	7.5 ± 1.2
**(n = 17)**	**4.9**	0.74 ± 0.09	11.7 ± 1.2	28.0 ± 3.2	94.8 ± 10.3	51.9 ± 5.6	26.7 ± 5.5	0.0079 ± 0.0010	9.1 ± 2.1	9.1 ± 1.9
	**5.9**	0.93 ± 0.13	14.7 ± 1.4	35.2 ± 3.9	105.2 ± 11.3	57.6 ± 5.8	35.4 ± 6.6	0.0089 ± 0.0012	10.8 ± 2.2	10.9 ± 2.0
	**Max**	2.66 ± 0.31	42.0 ± 4.4	100.0 ± 0.0	182.9 ± 8.1	100.0 ± 0.0	100.0 ± 0.0	0.0145 ± 0.0015	17.4 ± 2.1	18.6 ± 1.0
**Men**	**4.0**	0.86 ± 0.16	10.5 ± 1.2	24.1 ± 4.6	90.5 ± 14.8	50.0 ± 7.2	20.8 ± 9.7	0.0097 ± 0.0021	8.3 ± 1.2	8.1 ± 1.5
**(n = 17)**	**4.9**	1.01 ± 0.20	12.3 ± 1.4	28.3 ± 5.1	95.9 ± 13.3	53.0 ± 6.1	25.6 ± 8.1	0.0107 ± 0.0024	9.8 ± 1.6	9.5 ± 1.7
	**5.9**	1.27 ± 0.26	15.4 ± 1.8	35.4 ± 6.5	104.6 ± 14.3	57.8 ± 6.3	33.2 ± 8.4	0.0123 ± 0.0026	11.4 ± 1.5	11.2 ± 2.3
	**Max**	3.60 ± 0.54	44.3 ± 6.8	100.0 ± 0.0	180.7 ± 8.7	100.0 ± 0.0	100.0 ± 0.0	0.0200 ± 0.0031	17.4 ± 1.1	17.6 ± 1.8
**All**	**4.0**	0.75 ± 0.17	10.3 ± 1.2	24.1 ± 3.9	89.1 ± 13.2	49.0 ± 6.8	20.9 ± 8.1	0.0085 ± 0.0020	7.9 ± 1.3	7.8 ± 1.4
**(n = 34)**	**4.9**	0.88 ± 0.21	12.0 ± 1.3	28.1 ± 4.2	95.4 ± 11.7	52.4 ± 5.8	26.1 ± 6.8	0.0093 ± 0.0023	9.4 ± 1.9	9.3 ± 1.8
	**5.9**	1.10 ± 0.27	15.0 ± 1.7	35.3 ± 5.3	104.9 ± 12.7	57.7 ± 6.0	34.3 ± 7.5	0.0106 ± 0.0027	11.1 ± 1.9	11.1 ± 2.1
	**Max**	3.13 ± 0.65	43.2 ± 5.8	100.0 ± 0.0	181.8 ± 8.3	100.0 ± 0.0	100.0 ± 0.0	0.0173 ± 0.0037	17.4 ± 1.6	18.1 ± 1.5

### Regression equations

The constituents of the regression equations (y-intercept, slope and r-coefficient) and their absolute and relative differences between CEE and TE are presented for all participants of model 1 (only submaximal workloads) in Table 4 and model 2 (both submaximal and maximal workloads) in Table 5. There were no absolute or relative significant differences found for either the y-intercept or the slope in model 1 (n.s.). However, the r-coefficient was significantly higher for CEE than TE in both absolute, 0.022 ± 0.045 (P < 0.01), and relative numbers, 2.3 ± 4.6% (P < 0.01) (Table 4). In model 2, both the absolute and the relative differences between CEE and TE were significant for all regression coefficients. The absolute differences were 0.311 ± 0.532 (P < 0.01) for the y-intercept, -0.0025 ± 0.0036 (P < 0.01) for the slope and -0.004 ± 0.009 (P < 0.05) for the r-coefficient. The corresponding relative differences were 38.1 ± 59.8% (P < 0.01), -12.4 ± 16.3% (P < 0.001) and -0.4 ± 1.0% (P < 0.05)(Table 5).

**Table 4:** Regression equations of model 1, and the absolute and relative exercise mode differences (n = 34, means ± SD, (95% CI) and P-values).

	y-intercept	slope	r
**Cycle ergometer exercise (CEE)**	-1.356 ± 0.743 (-1.615 to -1.097)	0.0240 ± 0.0073 (0.0215 to 0.0266)	0.995 ± 0.008 (0.992 to 0.998)
**Treadmill exercise (TE)**	-1.364 ± 0.842 (-1.658 to -1.070)	0.0235 ± 0.0099 (0.0201 to 0.0270)	0.972 ± 0.045 (0.957 to 0.988)
**Abs. diff. CEE-TE**	0.009 ± 0.821 (-0.278 to 0.295)	0.0005 ± 0.0077 (-0.0022 to 0.0032)	0.022 ± 0.045 (0.007 to 0.038)
**P-values T-test/Wilcoxon**	0.952/0.952	0.738/0.611	0.007/0.008
**Rel. diff. CEE-TE (%)**	32.8 ± 180.1 (-30.0 to 95.7)	1.2 ± 35.8 (-11.3 to 13.7)	2.3 ± 4.6 (0.7 to 3.8)
**P-values T-test/Wilcoxon**	0.296/0.765	0.848/0.614	0.007/0.008

**Table 5:** Regression equations of model 2, and the absolute and relative exercise mode differences (n = 34, means ± SD, (95% CI) and P-values).

	y-intercept	slope	r
**Cycle ergometer exercise (CEE)**	-1.310 ± 0.609 (-1.523 to -1.098)	0.0236 ± 0.0060 (0.0215 to 0.0257)	0.994 ± 0.009 (0.991 to 0.997)
**Treadmill exercise (TE)**	-1.621 ± 0.559 (-1.816 to -1.426)	0.0261 ± 0.0058 (0.0241 to 0.0282)	0.998 ± 0.002 (0.997 to 0.999)
**Abs. diff. CEE-TE**	0.311 ± 0.532 (0.125 to 0.496)	-0.0025 ± 0.0036 (-0.0038 to -0.0013)	-0.004 ± 0.009 (-0.007 to -0.001)
**P-values T-test/Wilcoxon**	0.002/0.003	0.000/0.001	0.015/0.024
**Rel. diff. CEE-TE (%)**	38.1 ± 59.8 (17.2 to 58.9)	-12.4 ± 16.3 (-18.1 to -6.7)	-0.4 ± 1.0 (-0.8 to -0.1)
**P-values T-test/Wilcoxon**	0.001/0.000	0.000/0.000	0.015/0.024

### Estimation of oxygen uptake using the HR method

The estimated $\dot{V}O_{2}$ levels, based on the individual regression equations and seven individual HR values, derived from % of HRR, are presented for all participants in Tables 6 (model 1) and 7 (model 2). There were no significant absolute or relative differences between the estimated $\dot{V}O_{2}$ levels when comparing CEE with TE for any of the intensity levels between 25–85% of HRR (94.0–164.2 beats·min^-1^) in model 1. The $\dot{V}O_{2}$ differences between CEE and TE ranged from 0.05 ± 0.24 to 0.10 ± 0.51 L·min^-1^ and -0.4 ± 34.9 to 4.8 ± 17.1%, respectively (n.s.) (Table 6). When including the maximal workloads in the regression equations (model 2), the estimated $\dot{V}O_{2}$ differences between CEE and TE varied from 0.07 ± 0.26 to -0.05 ± 0.20 L·min^-1^ and 1.3 ± 40.3 to -2.7 ± 10.0%, respectively, (n.s.) in the intensity range of 25–65% of HRR (94.0–140.8 beats·min^-1^) (Table 7). At the two highest exercise intensities, 75 and 85% (152.5 and 164.2 beats·min^-1^), the $\dot{V}O_{2}$ levels were slightly lower during the cycle ergometer exercise than the treadmill exercise. These absolute $\dot{V}O_{2}$ differences were -0.07 ± 0.20 (P = T-test: 0.041, Wilcoxon: 0.071) and -0.10 ± 0.22 L·min^-1^ (P < 0.05), respectively, the corresponding relative differences were -3.7 ± 9.0% (P < 0.05) and -4.4 ± 8.6% (P < 0.05).

**Table 6:** Estimation of $\dot{V}O_{2}$ in model 1, and the absolute and relative exercise mode differences (n = 34, means ± SD, (95% CI) and P-values).

Exercise intensity							
	Very light–light		Moderate		Vigorous		
	25%HRR	35%HRR	45%HRR	55%HRR	65%HRR	75%HRR	85%HRR
**HR (beats·min**^**-1**^**)**	94.0 ± 7.5	105.7 ± 7.1	117.4 ± 6.8	129.1 ± 6.8	140.8 ± 6.8	152.5 ± 7.1	164.2 ± 7.5
**Estimation of** $\dot{V}O_{2}$ **(L·min**^**-1**^**)**							
**Cycle ergometer exercise (CEE)**	0.91 ± 0.31 (0.80 to 1.02)	1.19 ± 0.33 (1.07 to 1.30)	1.47 ± 0.37 (1.34 to 1.60)	1.75 ± 0.42 (1.60 to 1.89)	2.03 ± 0.48 (1.86 to 2.19)	2.30 ± 0.55 (2.11 to 2.50)	2.58 ± 0.62 (2.37 to 2.80)
**Treadmill exercise (TE)**	0.86 ± 0.26 (0.77 to 0.95)	1.13 ± 0.33 (1.01 to 1.24)	1.40 ± 0.42 (1.25 to 1.54)	1.67 ± 0.52 (1.49 to 1.85)	1.94 ± 0.62 (1.73 to 2.16)	2.21 ± 0.72 (1.96 to 2.47)	2.48 ± 0.83 (2.19 to 2.77)
**Abs. diff. CEE-TE (L·min**^**-1**^**)**	0.05 ± 0.24 (-0.03 to 0.13)	0.06 ± 0.22 (-0.02 to 0.14)	0.07 ± 0.25 (-0.02 to 0.15)	0.08 ± 0.29 (-0.03 to 0.18)	0.08 ± 0.36 (-0.04 to 0.21)	0.09 ± 0.43 (-0.06 to 0.24)	0.10 ± 0.51 (-0.08 to 0.28)
**P-values T-test/Wilcoxon**	0.207/0.164	0.127/0.135	0.116/0.118	0.142/0.149	0.183/0.158	0.226/0.185	0.265/0.228
**Rel. diff. CEE-TE (%)**	-0.4 ± 34.9 (-12.6 to 11.8)	3.7 ± 19.0 (-2.9 to 10.4)	4.6 ± 16.5 (-1.2 to 10.4)	4.8 ± 17.1 (-1.2 to 10.7)	4.7 ± 18.3 (-1.7 to 11.1)	4.6 ± 19.6 (-2.2 to 11.4)	4.5 ± 20.8 (-2.8 to 11.7)
**P-values T-test/Wilcoxon**	0.952/0.343	0.260/0.161	0.114/0.081	0.112/0.144	0.140/0.153	0.179/0.164	0.220/0.222

**Table 7:** Estimation of $\dot{V}O_{2}$ in model 2, and the absolute and relative exercise mode differences (n = 34, means ± SD, (95% CI) and P-values).

Exercise intensity							
	Very light–light		Moderate		Vigorous		
	25%HRR	35%HRR	45%HRR	55%HRR	65%HRR	75%HRR	85%HRR
**HR (beats·min**^**-1**^**)**	94.0 ± 7.5	105.7 ± 7.1	117.4 ± 6.8	129.1 ± 6.8	140.8 ± 6.8	152.5 ± 7.1	164.2 ± 7.5
**Estimation of** $\dot{V}O_{2}$ **(L·min**^**-1**^**)**							
**Cycle ergometer exercise (CEE)**	0.91 ± 0.33 (0.80 to 1.02)	1.19 ± 0.34 (1.07 to 1.30)	1.46 ± 0.36 (1.34 to 1.59)	1.74 ± 0.40 (1.60 to 1.88)	2.01 ± 0.45 (1.86 to 2.17)	2.29 ± 0.50 (2.11 to 2.46)	2.57 ± 0.56 (2.37 to 2.76)
**Treadmill exercise (TE)**	0.84 ± 0.29 (0.74 to 0.94)	1.15 ± 0.32 (1.04 to 1.26)	1.45 ± 0.36 (1.33 to 1.58)	1.76 ± 0.40 (1.62 to 1.90)	2.06 ± 0.45 (1.90 to 2.22)	2.36 ± 0.51 (2.19 to 2.54)	2.67 ± 0.56 (2.47 to 2.86)
**Abs. diff. CEE-TE (L·min**^**-1**^**)**	0.07 ± 0.26 (-0.02 to 0.16)	0.04 ± 0.23 (-0.04 to 0.12)	0.01 ± 0.21 (-0.06 to 0.09)	-0.02 ± 0.20 (-0.09 to 0.05)	-0.05 ± 0.20 (-0.12 to 0.02)	-0.07 ± 0.20 (-0.14 to 0.00)	-0.10 ± 0.22 (-0.18 to -0.03)
**P-values T-test/Wilcoxon**	0.136/0.114	0.339/0.215	0.777/0.590	0.615/0.955	0.189/0.379	0.041/0.071	0.009/0.011
**Rel. diff. CEE-TE (%)**	1.3 ± 40.3 (-12.8 to 15.3)	1.2 ± 21.8 (-6.4 to 8.8)	-0.3 ± 15.3 (-5.6 to 5.0)	-1.6 ± 11.9 (-5.8 to 2.5)	-2.7 ± 10.0 (-6.2 to 0.7)	-3.7 ± 9.0 (-6.8 to -0.5)	-4.4 ± 8.6 (-7.5 to -1.4)
**P-values T-test/Wilcoxon**	0.857/0.270	0.752/0.388	0.912/0.700	0.429/0.765	0.119/0.281	0.024/0.047	0.005/0.011

**Fig 2:**
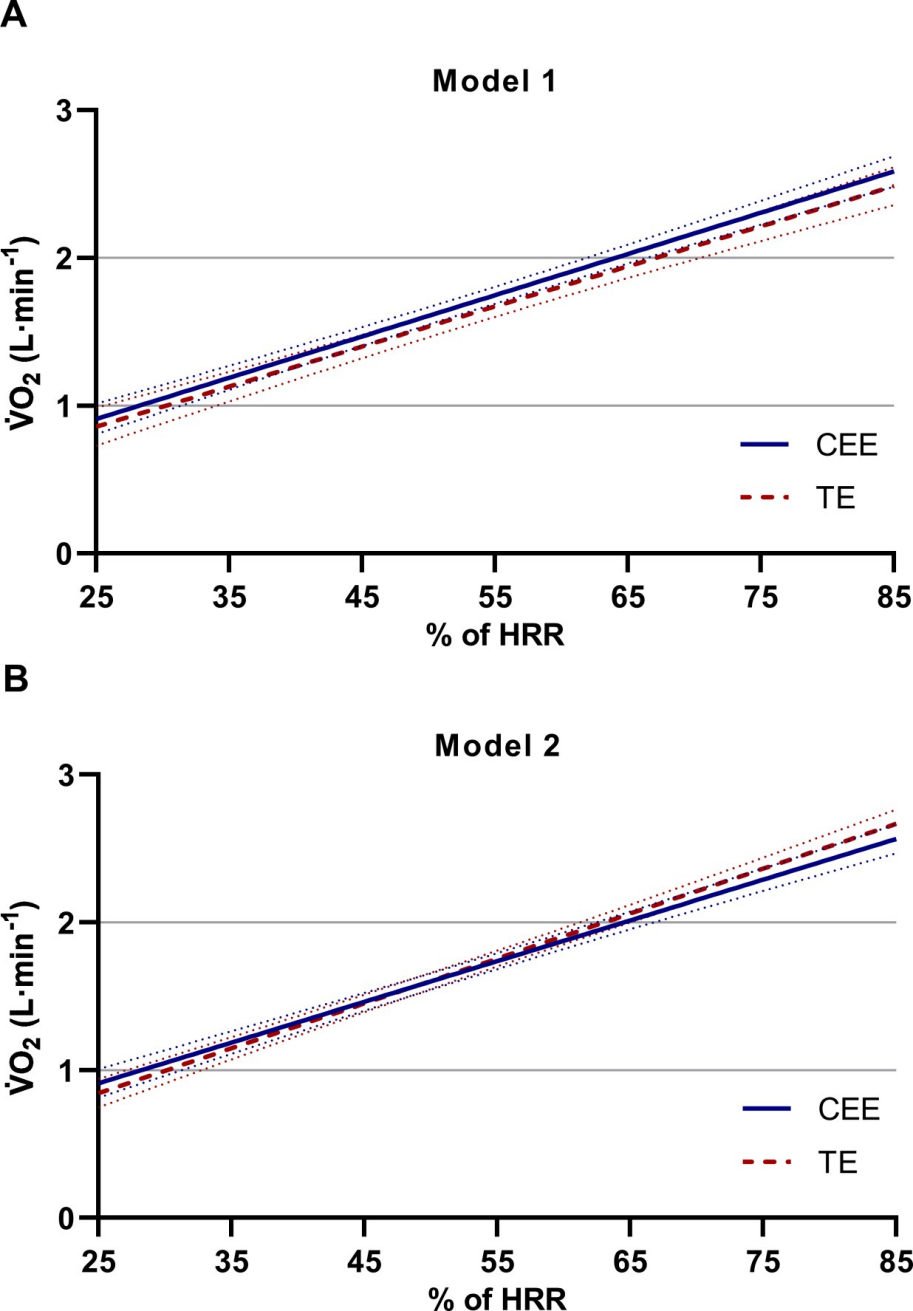
Estimated mean $\dot{V}O_{2}$ levels between 25–85% of HRR in cycle ergometer exercise (CEE) and treadmill exercise (TE) in model 1 and model 2. Based on all participants (n = 34) individual values. CEE: blue solid line. TE: red dashed line. The linear regression equations with 95% CI and r-coefficients were: Model 1 **(A)** y($\dot{V}O_{2}$CEE) = 0.210(0.042─0.377) + 0.0279(0.0251─0.0308) * x(%HRR), r-coefficient = 0.781, y($\dot{V}O_{2}$TE) = 0.178(-0.030─0.386) + 0.0271(0.0236─0.0307) * x(%HRR) and r-coefficient = 0.700. Model 2 **(B)** y($\dot{V}O_{2}$CEE) = 0.222(0.064─0.380) + 0.0276(0.0249─0.0303) * x(%HRR), r-coefficient = 0.795, y($\dot{V}O_{2}$TE) = 0.084(-0.072─0.241) + 0.0304(0.0277─0.0331) * x(%HRR) and r-coefficient = 0.825.

**Fig 3:**
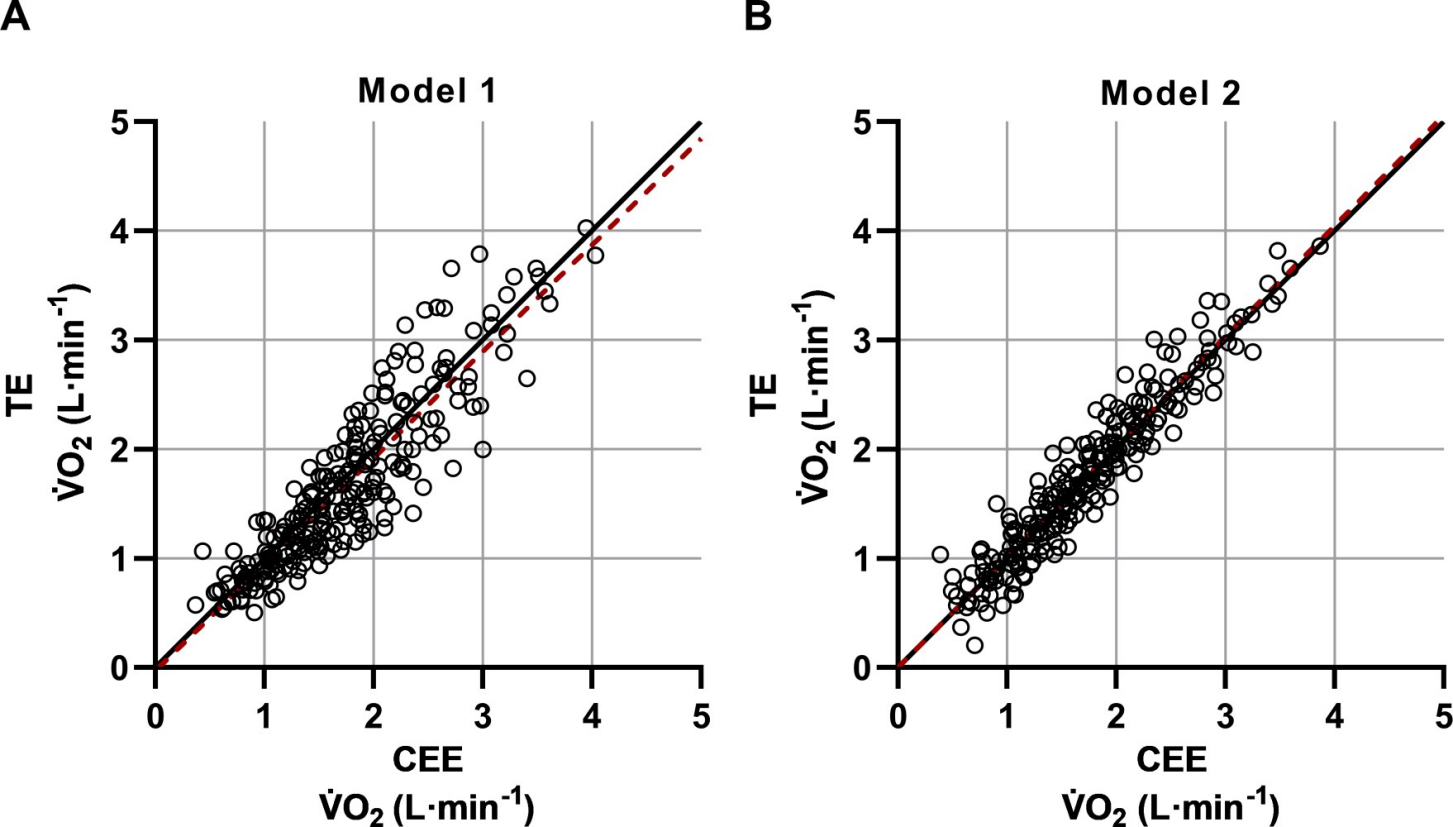
Comparison of the estimated individual $\dot{V}O_{2}$ values between cycle ergometer exercise (CEE) and treadmill exercise (TE) in model 1 and model 2. Based on all participants (n = 34), ranging between 25–85% of HRR. The overall linear regression equations (red dashed lines) with 95% CI and r-coefficients were: Model 1 **(A)** y($\dot{V}O_{2}$TE) = -0.033(-0.148─0.082) + 0.9753(0.9144─1.0362) * x($\dot{V}O_{2}$CEE) and r-coefficient = 0.899. Model 2 **(B)** y($\dot{V}O_{2}$TE) = -0.004(-0.081─0.073) + 1.0125(0.9714─1.0536) * x($\dot{V}O_{2}$CEE) and r-coefficient = 0.953.

**Fig 4:**
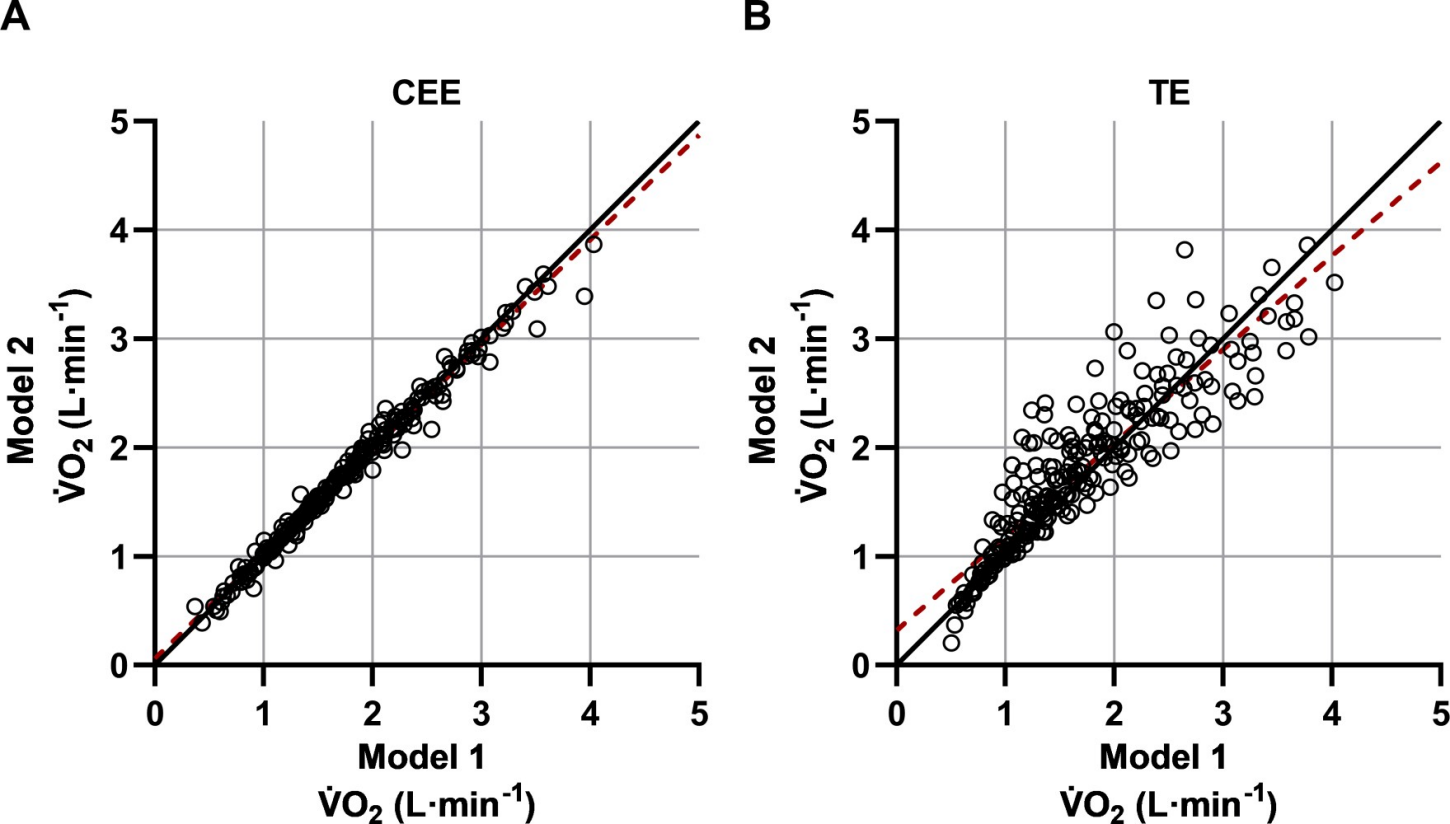
Comparison of the estimated individual $\dot{V}O_{2}$ values between model 1 and model 2 in cycle ergometer exercise (CEE) and treadmill exercise (TE). Based on all participants (n = 34), ranging between 25–85% of HRR. The overall linear regression equations (red dashed lines) with 95% CI and r-coefficients were: CEE **(A)** y($\dot{V}O_{2}$Model 2) = 0.056(0.028─0.084) + 0.9632(0.9483─0.9780) * x($\dot{V}O_{2}$Model 1) and r-coefficient = 0.993. TE **(B)** y($\dot{V}O_{2}$Model 2) = 0.318(0.223─0.413) + 0.8606(0.8091─0.9122) * x($\dot{V}O_{2}$Model 1) and r-coefficient = 0.906.

Illustrations of the estimated mean $\dot{V}O_{2}$ levels and the 95% confidence intervals, based on all individual values, are presented for all exercise intensity levels in Fig 2A (model 1) and 2B (model 2). Although these figures show that the average $\dot{V}O_{2}$ levels between the two exercise modalities differ to a very low extent, when instead comparing the individual $\dot{V}O_{2}$ values, the spreading between CEE and TE is generally greater in model 1 compared to model 2 (Fig 3A and 3B). Additionally, when comparing the same individual $\dot{V}O_{2}$ values between the two models within each exercise modality, the individual scattering is clearly less during the cycle ergometer exercise than the treadmill exercise (Fig 4A and 4B).

## Discussion

This is, to our knowledge, the first systematic study on the potential interchangeability of the HR method when estimating oxygen uptake in cycle ergometer exercise (CEE) and treadmill exercise (TE) with special reference to level walking. Another novel aspect was a search for pathways that can optimize the HR method. This was analysed through comparing two different models of $\mathrm{HR}‐\dot{V}O_{2}$ relationships. Model 1 consisted of three submaximal steady state workloads of ergometer cycling or level walking on the treadmill, while model 2 included one additional workload of maximal exercise in CEE and TE.

In order to apply the HR method, the development of regression equations is a necessary first step. A number of previous studies [18, 20, 51, 52] investigating issues related to the HR method have primarily based their evaluations on separate regression coefficients. This division of analyses can, however, lead to misinterpretations of the consequences of the HR method. We have therefore instead applied the HR method by estimating different levels of $\dot{V}O_{2}$ based on a broad range of HR values, and combined it with analyses of ingredients from the regression equations.

The main results are that only small, and in most cases (24 of 28) non-significant differences in estimated $\dot{V}O_{2}$ levels (range of relative means: -4.4 to 4.8%) were noted between CEE and TE when applying the HR method at intensity levels between 25–85% of HRR in both models. When interpreting the results, we observe that these relative mean $\dot{V}O_{2}$ differences between CEE and TE are of the same order of magnitude as the relative test-retest differences (range of means: 1.0–3.6%) of the HR method using ergometer cycling [21, 22]. At the same time, rather large standard deviations and confidence intervals were occasionally seen among the $\dot{V}O_{2}$ differences between the two exercise modalities in both models. Thus, there may be some undetected exercise mode differences due to the limited power of these results. If there are any further significant differences, they are, however, most likely of rather low magnitude and will therefore be of minor importance at the group level, but they may still be critical for validity at the individual level.

The absence of significant differences in estimated $\dot{V}O_{2}$ levels between submaximal ergometer cycling and level walking (model 1; Table 6) is in line with the only previous study that we can find that has specifically compared the $\mathrm{HR}‐\dot{V}O_{2}$ relationship of ergometer cycling with horizontal treadmill exercise [26]. Although, Berggren and Hohwü Christensen [26] did not apply the HR method, they observed similar $\dot{V}O_{2}$ levels at equivalent HR values in ergometer cycling and walking in one trained man.

Whether the HR-method is interchangeable between ergometer cycling and other forms of treadmill exercise, such as level running, as well as walking or running using inclination are also relevant questions. After studying previous literature [18, 26, 53–55], our analyses of appropriate studies [18, 54, 55], indicate that the HR method, based on ergometer cycling, is, at group level, interchangeable with level treadmill running, but not with treadmill exercise using inclination. For instance, our analysis of Lafortuna, Agosti [18] demonstrated between 16–24% (obese women) and 14–19% (normal weight women) lower $\dot{V}O_{2}$ levels at equivalent HR values during submaximal ergometer cycling when compared with treadmill walking using work load increments of both increased speed and inclination. All our analyses, results and supplementary texts in this literature survey can be found in the supporting information in S1 Discussion. The overall picture indicates a clear need for further studies of these matters.

Issues of optimization of the HR method protrude when moving the focus from effects on mean values (cf. Fig 2) to effects on individual values (cf. Figs 3 and 4). It is notable that the deviances between the two exercise modalities are generally greater in model 1 than in model 2 (Fig 3 hin and 3B). Thus, the differences between CEE and TE seem to be reduced when the maximal workloads are added to the $\mathrm{HR}‐\dot{V}O_{2}$ relationships. Interestingly, when comparing model 1 with model 2 within CEE, in contrast to TE, the values are well aligned along the line of identity (cf. Fig 4A and 4B). Thus, the HR method in CEE, in itself, does not gain anything through adding maximal exercise. Thereby it cannot explain the reduction in spreading when comparing CEE and TE in model 1 with model 2 (Fig 3A and 3B). It is more likely that it is due to changes between model 1 and model 2 within TE, as indicated by a higher r-coefficient in model 2 (r = 0.998) compared to model 1 (r = 0.972) (Tables 4 and 5).

How can this be understood? We think that a reasonable starting point in explaining the overall pattern of differences between CEE and TE, as well as between the two models, is to view them in relation to the positions and widths of the submaximal measurement ranges applied, as well as their distances to the maximal HR and $\dot{V}O_{2}$ values. In model 1, the submaximal ergometer cycling was performed at average HR levels ranging between 103.3–148.7 beats·min^-1^ (based on all participants), whereas the corresponding range of level walking was between 89.1–104.9 beats·min^-1^. The rather narrow submaximal range in TE, as compared to CEE, increases the sensitivity to variations in HR and $\dot{V}O_{2}$ values on the linear regression equations. This is mirrored in the significantly different r-coefficients in ergometer cycling (0.995) and level walking (0.972) for model 1 (Table 4). An additional explanation to this instability in the individual submaximal TE values is that activities of light intensity, such as sedentary activities and slow walking, have been demonstrated to generate more varying $\mathrm{HR}‐\dot{V}O_{2}$ relationships than activities of moderate exercise intensity [11, 56, 57]. The wide gap between the submaximal and maximal HR and $\dot{V}O_{2}$ values add further to the instability within the TE models. To include a broader range of submaximal measurement points in level walking may assist in curing the instability noted in this study, contribute to optimizing the HR method for level walking, and thereby enhance its interchangeability with ergometer cycling.

As stated in the Introduction, a number of methodological issues related to the HR method need to be sorted out. Previously we have studied the reproducibility of the HR method in ergometer cycling [21, 22]. In the present study we have evaluated the interchangeability of the HR method between two exercise modalities, and searched for ways to optimize the method. In future studies, we will evaluate the external validity of the HR method in relation to exercise in field conditions. It is, in this context, important to gain knowledge about if slow components of HR and $\dot{V}O_{2}$ kinetics [48, 58, 59] lead to drifts in $\mathrm{HR}‐\dot{V}O_{2}$ relationships with time, and in such cases under which conditions. If, for example, HR starts to increase disproportionately more than $\dot{V}O_{2}$, then this must be compensated for as part of the HR method. One way to evaluate a need for such a correction would be to study each individual in the laboratory during prolonged exercise at the average intensity that is anticipated in the measurement context of interest. Additionally, this matter deserves to be studied under conditions of prolonged intermittent exercise that mimic applied measurement conditions. In fact, ocular inspections of HR and $\dot{V}O_{2}$ recordings during active commuting point in the direction of that if slow components exist, they increase to the same relative degree in HR and $\dot{V}O_{2}$ [41].

In conclusion, the current study demonstrates that the HR methods based on submaximal ergometer cycling and level walking are interchangeable for estimating mean $\dot{V}O_{2}$ levels between very light and vigorous exercise intensities corresponding to 25–85% of HRR. Fundamentally, the same finding is observed when including maximal exercise in the $\mathrm{HR}‐\dot{V}O_{2}$ relationships. Finally, the inter-individual variation in $\dot{V}O_{2}$ between ergometer cycling and treadmill exercise is reduced when using the HR method based on both submaximal and maximal workloads.

## Supporting information

S1 Methods The Physically Active commuting in Greater Stockholm Questionnaire 1 (PACS Q1). The original version in Swedish.
(DOC)

S2 Methods The Physically Active commuting in Greater Stockholm Questionnaire 1 (PACS Q1). The original version in Swedish translated into English.
(DOC)

S1 Discussion Analyses of previous literature using the HR method for estimating oxygen uptake in ergometer cycling, and treadmill walking and running. (DOCX)
