# Supplementary material for: Are heart rate methods based on ergometer cycling and level treadmill walking interchangeable?
Source: PLoS One. 2020 Aug 6;15(8):e0237388. doi: 10.1371/journal.pone.0237388 (PMC7410327; doi:10.1371/journal.pone.0237388)
Supplement: S1 Discussion — (DOCX) [file pone.0237388.s003.docx]

**Analyses of previous literature using the HR method for estimating oxygen uptake in ergometer cycling, and treadmill walking and running**

In order to expand our main examination of the interchangeability between ergometer cycling and level treadmill walking when using the HR method, we compared our results with previous studies that have reported data for HR-V̇O_2_ relationships in both cycle ergometer exercise and various forms of treadmill exercise [18, 26, 53-55]. As a part of this, we made our own analyses based on individual or average HR-V̇O_2_ data from earlier studies [18, 54, 55], by using the HR method for estimating V̇O_2_ at four selected HR values; 100, 125, 150 and 175 beats·min^-1^. The focus with this supplementary examination has been to investigate if the HR method, based on ergometer cycling, is valid for estimating V̇O_2_ during level treadmill running as well as treadmill walking or running using inclinations.

**Is the HR method based on ergometer cycling** **applicable for estimating oxygen uptake during level treadmill running?**

The single-subject study by Berggren and Hohwü Christensen (26) implies this, since they observed similar V̇O_2_ levels in ergometer cycling as compared to level walking and running on treadmill at equal HR values. Moreover, our analysis of Abrantes, Sampaio (55) average data, using 20 physically active male subjects, demonstrated only between 1-7% lower V̇O_2_ levels for ergometer cycling comparing with level running (Tables 8 and 9). Thus, the overall minor V̇O_2_ differences indicate that the HR method, established in ergometer cycling, is valid during level running on treadmill. However, since this issue has not been specifically investigated, it is recommended that level treadmill running is further studied and compared with level walking and ergometer cycling.

**Analysis of Abrantes, Sampaio (55)**

**Table 8. Calculated regression equations based on the mean values (n = 20) in Abrantes, Sampaio (55).**

|  | **y-intercept** | **slope** | **r** |
| --- | --- | --- | --- |
| **Cycle ergometer exercise (CEE)** | -1,115 | 0,0252 | 0,999 |
| **Treadmill exercise (TE)** | -0,930 | 0,0244 | 0,994 |

**Table 9. Estimation of V̇O_2_, using the HR method at four selected HR values and the regression**

**equations (in Table 8), and the absolute and relative exercise mode differences.**

|  | **HR values (beats·min^-1^)** | | | |
| --- | --- | --- | --- | --- |
|  | **100** | **125** | **150** | **175** |
| **Estimation of V̇O_2_ (L·min^-1^)** | | | | |
| **Cycle ergometer exercise (CEE)** | 1.41 | 2.04 | 2.67 | 3.30 |
|  |  |  |  |  |
| **Treadmill exercise (TE)(inclination 0˚)** | 1.51 | 2.12 | 2.73 | 3.34 |
|  |  |  |  |  |
| **Abs. diff.**  **CEE-TE (L·min^-1^)** | -0.11 | -0.09 | -0.06 | -0.05 |
|  |  |  |  |  |
| **Rel. diff.**  **CEE-TE (%)** | -7 | -4 | -2 | -1 |
|  |  |  |  |  |

**Is the HR method based on ergometer cycling** **applicable for estimating oxygen uptake during treadmill walking and running using inclinations?**

Our analysis of Lafortuna, Agosti (18), based on its mean regression equations contradicts this fact, since it showed between 16-24% lower V̇O_2_ levels during ergometer cycling in comparison to treadmill walking in 15 obese women and 14-19% lower V̇O_2_ levels in six normal weight women (Tables 10-11). Workload increments of both speed and increasing inclination (0-2.3˚ in obese/0-3.7˚ in normal weight) were used. Both the ergometer cycling and the treadmill walking were carried out through submaximal steady state workloads. Moreover, Lafortuna, Agosti (18) compared the various exercise modalities’ regression coefficients and found significant differences (P < 0.001) between the y-intercepts and slopes in the obese women and among the y-intercepts in the normal weight women.

In another study, results in the same direction have also been hinted. Hermansen and Saltin (53) observed slightly significantly higher HR values (P < 0.05) in submaximal treadmill exercise using a constant inclination of 3˚ uphill in comparison to ergometer cycling at equal metabolic rates, when investigating eight endurance trained males (4-6 beats·min^-1^) and six untrained male students (8-14 beats·min^-1^). In a subsequent study by Hermansen, Ekblom (54), their previous results were confirmed as well as in our analysis of their results. Our analysis of Hermansen, Ekblom (54), based on individual paired values of HR and V̇O_2_, demonstrated no significant exercise mode differences among the regression coefficients (Tables 12-13). This agrees with the results of Hermansen, Ekblom (54). However, our analyses using the HR method indicated between 4-17% (only submaximal workloads) and 6-20% (submaximal and maximal workloads) lower V̇O_2_ levels for ergometer cycling comparing with treadmill running (Tables 14-15). The HR-V̇O_2_ relationships, including only submaximal workloads (model 1) were based on five healthy male subjects, while the HR-V̇O_2_ relationships, including both submaximal and maximal workloads (model 2) were based on seven healthy male subjects. The differences between the exercise modalities were only significant (P < 0.05) in three of 16 V̇O_2_ comparisons between CEE and TE (including both absolute and relative differences in model 1 and model 2). However, in the article [54] it is undefined if inclination has been used and perhaps contributed to the rather higher V̇O_2_ levels during treadmill running.

Taken together, this literature review indicates that there are differences in the estimated V̇O_2_ levels when using the HR method in ergometer cycling and horizontal treadmill exercise as opposed to treadmill exercise using inclinations. It is possible that the vertical movement of the entire body weight causes these differences. Nevertheless, this issue requires further investigation. Furthermore, if this hypothesis is correct, that there are differences in oxygen cost at equivalent HR values, it will obviously also affect the usefulness of the HR method to estimate V̇O_2_ for walking and running in field conditions with varying topography.

**Analysis of Lafortuna, Agosti (18)**

**Table 10. Estimation of V̇O_2_, using the HR method at four selected HR values and the regression equations from the obese women (n = 15) in Lafortuna, Agosti (18), and the absolute and relative exercise mode differences.**

|  | **HR values (beats·min^-1^)** | | | |
| --- | --- | --- | --- | --- |
|  | **100** | **125** | **150** | **175** |
| **Estimation of V̇O_2_ (L·min^-1^)** | | | | |
| **Cycle ergometer**  **exercise (CEE)** | 0.83 | 1.29 | 1.74 | 2.20 |
|  |  |  |  |  |
| **Treadmill exercise (TE) (inclination 0-2.3˚)** | 0.97 | 1.55 | 2.14 | 2.72 |
|  |  |  |  |  |
| **Abs. diff.**  **CEE-TE (L·min^-1^)** | -0.13 | -0.26 | -0.39 | -0.52 |
|  |  |  |  |  |
| **Rel. diff.**  **CEE-TE (%)** | -16 | -20 | -22 | -24 |

**Table 11. Estimation of V̇O_2_, using the HR method at four selected HR values and the regression equations from the normal weight women (n = 6) in Lafortuna, Agosti (18), and the absolute and relative exercise mode differences.**

|  | **HR values (beats·min^-1^)** | | | |
| --- | --- | --- | --- | --- |
|  | **100** | **125** | **150** | **175** |
| **Estimation of** **V̇O_2_ (L·min^-1^)** | | | | |
| **Cycle ergometer**  **exercise (CEE)** | 0.64 | 0.99 | 1.33 | 1.68 |
|  |  |  |  |  |
| **Treadmill exercise (TE) (inclination 0-3.7˚)** | 0.77 | 1.15 | 1.53 | 1.91 |
|  |  |  |  |  |
| **Abs. diff.**  **CEE-TE (L·min^-1^)** | -0.12 | -0.16 | -0.19 | -0.23 |
|  |  |  |  |  |
| **Rel. diff.**  **CEE-TE (%)** | -19 | -16 | -15 | -14 |

**Analysis of Hermansen, Ekblom (54)**

This analysis is based on individual paired values of HR and V̇O_2_. Subjects who had three or four measurement points of both cycle ergometer exercise and treadmill exercise were included. Two different models of HR-V̇O_2_ relationships were created for each form of exercise. Model 1 included only submaximal workloads (based on five subjects), while model 2 included also one additional workload of maximal exercise (based on seven subjects).

**Table 12. Calculated regression equations of model 1, and the absolute and relative exercise mode differences (n = 5, means ± SD, (95 % CI) and P-values).**

|  | **y-intercept** | **slope** | **r** |
| --- | --- | --- | --- |
| **Cycle ergometer exercise (CEE)** | -1.705 ± 0.260 (-2.027 to -1.382) | 0.0282 ± 0.0045 (0.0226 to 0.0337) | 0.998 ± 0.002 (0.995 to 1.001) |
| **Treadmill exercise (TE)** | -1.498 ± 0.569 (-2.204 to -0.792) | 0.0279 ± 0.0080 (0.0180 to 0.0378) | 0.994 ± 0.008 (0.984 to 1.004) |
| **Abs. diff. CEE-TE** | -0.206 ± 0.421 (-0.728 to 0.316) | 0.0003 ± 0.0041 (-0.0048 to 0.0053) | 0.004 ± 0.009 (-0.008 to 0.016) |
| **P-values T-test/Wilcoxon** | 0.335/0.345 | 0.893/0.893 | 0.391/0.465 |
| **Rel. diff. CEE-TE (%)** | -13.4 ± 25.0 (-44.5 to 17.6) | 2.3 ± 14.8 (-16.1 to 20.6) | 0.4 ± 0.9 (-0.8 to 1.6) |
| **P-values T-test/Wilcoxon** | 0.296/0.225 | 0.747/0.893 | 0.391/0.465 |

**Table 13. Calculated regression equations of model 2, and the absolute and relative exercise mode differences (n = 7, means ± SD, (95 % CI) and P-values).**

|  | **y-intercept** | **slope** | **r** |
| --- | --- | --- | --- |
| **Cycle ergometer exercise (CEE)** | -2.285 ± 0.647 (-2.884 to -1.686) | 0.0328 ± 0.0054 (0.0278 to 0.0378) | 0.992 ± 0.008 (0.985 to 0.999) |
| **Treadmill exercise (TE)** | -2.188 ± 0.747 (-2.879 to -1.498) | 0.0334 ± 0.0072 (0.0267 to 0.0401) | 0.986 ± 0.022 (0.965 to 1.007) |
| **Abs. diff. CEE-TE** | -0.097 ± 0.479 (-0.540 to 0.347) | -0.0007 ± 0.0034 (-0.0038 to 0.0024) | 0.006 ± 0.022 (-0.014 to 0.026) |
| **P-values T-test/Wilcoxon** | 0.613/0.499 | 0.616/0.612 | 0.496/0.686 |
| **Rel. diff. CEE-TE (%)** | -3.1 ± 26.9 (-28.0 to 21.8) | -1.7 ± 11.2 (-12.0 to 8.6) | 0.6 ± 2.2 (-1.4 to 2.6) |
| **P-values T-test/Wilcoxon** | 0.772/0.499 | 0.706/0.735 | 0.497/0.686 |

**Table 14. Estimation of V̇O_2_ in model 1, using the HR method at four selected HR values and the calculated individual regression equations, and the absolute and relative exercise mode differences (n = 5, means ± SD, (95 % CI) and P-values).**

|  | **HR values (beats·min^-1^)** | | | |
| --- | --- | --- | --- | --- |
|  | **100** | **125** | **150** | **175** |
| **Estimation of V̇O_2_ (L·min^-1^)** | |  |  |  |
| **Cycle ergometer**  **exercise (CEE)** | 1.11 ± 0.32  (0.71 to 1.51) | 1.82 ± 0.42  (1.30 to 2.33) | 2.52 ± 0.52  (1.88 to 3.16) | 3.22 ± 0.62  (2.45 to 4.00) |
| **Treadmill exercise**  **(TE)(inclination?)** | 1.29 ± 0.33  (0.88 to 1.71) | 1.99 ± 0.51  (1.36 to 2.62) | 2.69 ± 0.69  (1.82 to 3.55) | 3.38 ± 0.89  (2.28 to 4.49) |
| **Abs. diff.**  **CEE-TE (L·min^-1^)** | -0.18 ± 0.11  (-0.31 to -0.05) | -0.17 ± 0.15  (-0.36 to 0.01) | -0.17 ± 0.23  (-0.45 to 0.12) | -0.16 ± 0.33  (-0.56 to 0.24) |
| **P-values**  **T-test/Wilcoxon** | 0.020/0.043 | 0.060/0.078 | 0.181/0.176 | 0.331/0.225 |
|  |  |  |  |  |
| **Rel. diff.**  **CEE-TE (%)** | -17.2 ± 11.9  (-32.0 to -2.3) | -9.0 ± 9.4  (-20.7 to 2.6) | -5.7 ± 10.0  (-18.1 to 6.7) | -3.9 ± 10.7  (-17.3 to 9.4) |
| **P-values**  **T-test/Wilcoxon** | 0.032/0.043 | 0.098/0.080 | 0.269/0.345 | 0.459/0.500 |
|  |  |  |  |  |

**Table 15. Estimation of V̇O_2_ in model 2, using the HR method at four selected HR values and the calculated individual regression equations, and the absolute and relative exercise mode differences (n = 7, means ± SD, (95 % CI) and P-values).**

|  | **HR values (beats·min^-1^)** | | | |
| --- | --- | --- | --- | --- |
|  | **100** | **125** | **150** | **175** |
| **Estimation of V̇O_2_ (L·min^-1^)** | |  |  |  |
| **Cycle ergometer**  **exercise (CEE)** | 0.99 ± 0.33  (0.69 to 1.30) | 1.81 ± 0.35  (1.49 to 2.13) | 2.63 ± 0.42  (2.24 to 3.01) | 3.45 ± 0.51  (2.98 to 3.92) |
| **Treadmill exercise**  **(TE)(inclination?)** | 1.15 ± 0.36  (0.82 to 1.49) | 1.99 ± 0.43  (1.59 to 2.39) | 2.83 ± 0.56  (2.31 to 3.34) | 3.66 ± 0.70  (3.01 to 4.31) |
| **Abs. diff.**  **CEE-TE (L·min^-1^)** | -0.16 ± 0.22  (-0.37 to 0.04) | -0.18 ± 0.20  (-0.37 to 0.00) | -0.20 ± 0.21  (-0.39 to 0.00) | -0.21 ± 0.25  (-0.45 to 0.02) |
| **P-values**  **T-test/Wilcoxon** | 0.100/0.091 | 0.054/0.063 | 0.048/0.043 | 0.065/0.063 |
|  |  |  |  |  |
| **Rel. diff.**  **CEE-TE (%)** | -19.7 ± 29.7  (-47.2 to 7.7) | -9.8 ± 12.5  (-21.4 to 1.7) | -7.0 ± 8.3  (-14.7 to 0.7) | -5.6 ± 7.1  (-12.2 to 1.0) |
| **P-values**  **T-test/Wilcoxon** | 0.129/0.128 | 0.083/0.063 | 0.067/0.063 | 0.082/0.091 |
|  |  |  |  |  |
